# Congenital Tooth Agenesis and Risk of Early-Onset Cancer

**DOI:** 10.1001/jamanetworkopen.2024.0365

**Published:** 2024-03-15

**Authors:** Saga Elise Eiset, Jeremy Schraw, Gitte Vrelits Sørensen, Pernille Axél Gregersen, Sonja A. Rasmussen, Cecilia H. Ramlau-Hansen, Philip J. Lupo, Henrik Hasle

**Affiliations:** 1 Department of Clinical Medicine, Aarhus University, Aarhus, Denmark; 2 Department of Pediatrics and Adolescent Medicine, Aarhus University Hospital, Aarhus, Denmark; 3 Department of Child and Adolescent Psychiatry, Aarhus University Hospital, Aarhus, Denmark; 4 Section of Hematology-Oncology, Department of Pediatrics, Baylor College of Medicine, Houston, Texas; 5 Department of Clinical Genetics, Aalborg University Hospital, Aalborg, Denmark; 6 Department of Clinical Genetics, Aarhus University Hospital, Aarhus, Denmark; 7 Johns Hopkins University School of Medicine, Baltimore, Maryland; 8 Department of Public Health–Epidemiology, Aarhus University, Aarhus, Denmark; 9 Section of Hematology-Oncology, Department of Pediatrics, Baylor College of Medicine, Houston, Texas

## Abstract

**Importance:**

There is some evidence that tooth agenesis (congenital absence of 1 or more teeth) is associated with cancer risk, especially carcinomas of the colon and ovaries, but results of previous studies are conflicting, and associations have not yet been evaluated in a population-based setting.

**Objective:**

To examine the association between tooth agenesis and specific cancer types before 40 years of age.

**Design, Setting, and Participants:**

This population-based cohort study used linking data from nationwide registries in Denmark to assess all Danish live-born singletons born from January 1, 1977, to December 31, 2018, and followed up for up to 40 years. Data were analyzed from January through June 2023.

**Exposure:**

Tooth agenesis as documented by the Danish Central Registry of Odontology (Danish municipal pediatric dental care) from January 1, 1988, to December 31, 2018, and from hospital encounters in the Danish National Patient Registry within the entire study period.

**Main Outcome and Measures:**

The primary outcome was first cancer diagnosis before 40 years of age obtained from the Danish Cancer Registry. Associations between tooth agenesis and specific cancers were estimated by Cox proportional hazards regression as hazard ratios (HRs) with 95% CIs. Analyses were split into age groups: younger than 1 year, 1 to younger than 3 years, 3 to younger than 10 years, 10 to younger than 20 years, 20 to younger than 30 years, and 30 to younger than 40 years. Associations with nonsyndromic tooth agenesis were evaluated after exclusion of individuals with known syndromes.

**Results:**

Among 2 501 715 included individuals (1 284 292 [51.3%] male), 70 288 (2.8%) had a diagnosis of tooth agenesis (mean [SD] age at diagnosis, 13.2 [4.1] years) and 26 308 (1.1%) had a diagnosis of early-onset cancer within the study period; 778 individuals had co-occurrence of tooth agenesis and cancer. Overall, tooth agenesis was positively associated with several cancer types, including neuroblastoma (age 1 to <3 years; HR, 4.20; 95% CI, 2.24-7.88), nephroblastoma (age 1 to <3 years; HR, 4.59; 95% CI, 2.37-8.91), hepatoblastoma (age 1 to <3 years; HR, 7.10; 95% CI, 2.70-18.68), osteosarcoma (age 10 to <20 years; HR, 2.19; 95% CI, 1.11-4.32), colorectal carcinomas (age 30 to <40 years; HR, 2.81; 95% CI, 1.38-5.71), and carcinomas of bladder (age 20 to <30 years; HR, 3.35; 95% CI, 1.35-8.30).

**Conclusions and Relevance:**

This cohort study found associations between congenital tooth agenesis and several cancer types, from childhood to early adulthood. Further evaluation of these associations is needed to assess possible clinical implications.

## Key Points

**Question:**

Is tooth agenesis associated with early-onset cancer?

**Findings:**

In this population-based cohort study of 2.5 million live-born singletons with up to 40 years of follow-up, tooth agenesis was positively associated with several cancer types, including neuroblastoma, nephroblastoma, and hepatoblastoma in childhood; osteosarcoma in adolescence; and colorectal carcinomas and carcinomas of the bladder in young adulthood.

**Meaning:**

These findings suggest that tooth agenesis is associated with specific cancer types, particularly in early childhood and early adulthood; further evaluation of these associations is needed to assess possible clinical implications.

## Teaser

This cohort study of live-born singletons in Denmark examines whether tooth agenesis is associated with early-onset cancer.

## Introduction

Congenital absence of 1 or more teeth (tooth agenesis) is common, with an estimated prevalence of 7.8% in Danish school children.^1^ In rare cases, tooth agenesis presents as part of a syndrome (syndromic tooth agenesis); it is a cardinal feature of ectodermal dysplasia^2^ and a possible feature of Down syndrome, Sotos syndrome, tuberous sclerosis complex, and other genetic syndromes.^3,4^ Several causative genes have also been identified for isolated (nonsyndromic) tooth agenesis, which can present with variable phenotypes, even within families.^3,5^ Variants in some genes, such as *WNT10A* (OMIM 150400) and *EDA* (OMIM 300451), can present as both syndromic and nonsyndromic tooth agenesis.^4^

**Figure 1.**
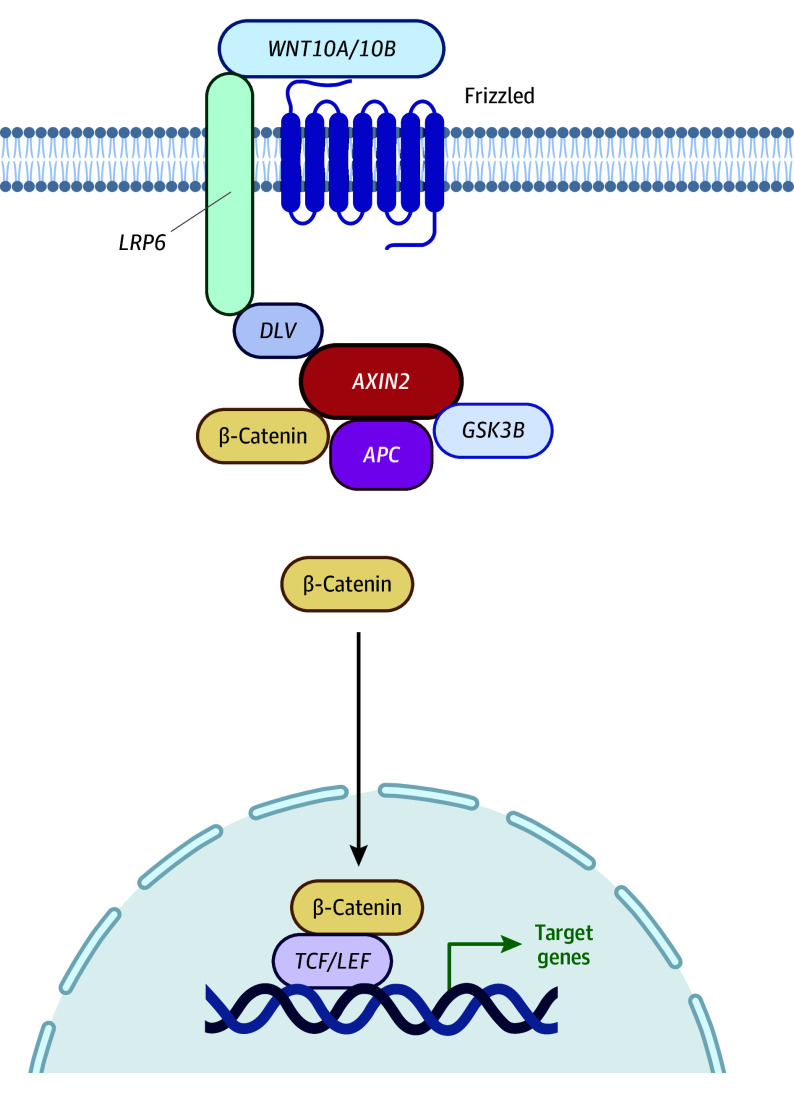
Wingless/Integrated (Wnt) Pathway in Tooth Formation *WNT10A/10B* activates the signaling cascade that upregulates β-catenin through inhibition of the *AXIN2/APC* complex. The *AXIN2* and *APC* genes are also known cancer predisposition genes. Created with BioRender software (BioRender).

Previous reports have suggested that individuals with tooth agenesis may have an increased risk of developing certain cancer types, as some of the cellular signaling pathways important for tooth formation also play a role in cancer development.^5,6,7,8,9,10^ This is particularly evident for the wingless/integrated (Wnt) pathway (Figure 1), which involves genes related to both tooth agenesis and cancer predisposition.^11^ Accordingly, pathogenic variants in the gene *AXIN2* (OMIM 604025) cause oligodontia-colorectal cancer syndrome, a rare cancer predisposition syndrome that presents with congenital tooth agenesis and primarily colorectal cancer in younger adults.^12^ In *APC* (OMIM 611731)–related familial adenomatous polyposis, colorectal cancers may present in adolescence, and dental anomalies are found in up to 75% of patients.^13^ Other genes of the Wnt and related pathways are known to be associated with tooth agenesis but have not yet been linked to cancer risk. Building from this evidence, we examine the hypothesis that tooth agenesis is associated with early-onset cancer using a population-based cohort design with up to 40 years of follow-up.

## Methods

This cohort study is registered at Aarhus University, Denmark, and has been performed in accordance with Danish law and European General Data Protection Regulation. Data access was approved by the Danish Health Data Authority. According to Danish law, informed consent is not required for register-based studies. A detailed description of registries is available in eMethods 1 in Supplement 1. The study followed the Strengthening the Reporting of Observational Studies in Epidemiology (STROBE) reporting guideline for cohort studies.^14^

### Study Population

**Figure 2.**
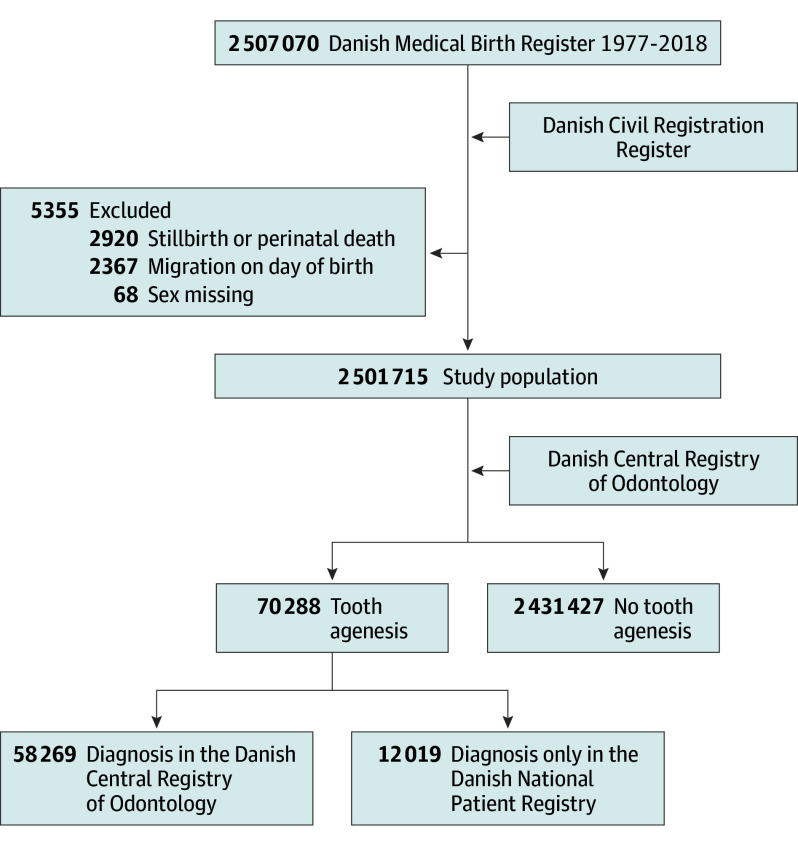
Study Cohort Flowchart

Singleton live births in Denmark from January 1, 1977, to December 31, 2018, were included from the Danish Medical Birth Register, with information on gestational age, birth weight, and maternal age.^15^ Date of birth, sex, and migration dates were obtained from the Danish Civil Registration Register,^16^ which also contains unique civil registration numbers used for register linkage. Individuals were excluded if date of birth was equal to either date of death or date of first emigration (Figure 2).

### Tooth Agenesis Diagnosis

Tooth agenesis was defined as missing 1 or more teeth from birth, excluding third molars. No differentiation among phenotypes was made because the correlation between agenesis of deciduous teeth (rare) and the permanent dentition (common) is very high,^17^ and number of missing teeth in heritable tooth agenesis can vary considerably even within families.^3,5^ Data on tooth agenesis were obtained from 2 different national registries. The Danish Central Registry of Odontology^18^ contains data from the municipal pediatric dental care system, which is free of charge and easily accessible for all Danish children (until 18 years of age during the study period). Registration of congenital tooth agenesis began in 1988.

Because the Danish Central Registry of Odontology did not register tooth agenesis in the entire study period, additional information was obtained from the Danish National Patient Registry.^19^ This registry contains discharge information from Danish nonpsychiatric hospitals from 1977 and outpatient hospital encounters, specialty clinics, and emergency departments from 1994. Diagnoses are registered according to the Danish adaptations of the *International Classification of Diseases, Eighth Revision* (*ICD-8*) from 1977 through 1993 and *International Statistical Classification of Diseases and Related Health Problems, Tenth Revision* (*ICD-10*) from 1994 onward. Tooth agenesis *ICD-8* code 520.09 and *ICD-10* code K00.0 (including subcodes) were included. This definition excludes acquired absence of teeth, which is coded elsewhere (*ICD-8* code 525.09 and *ICD-10* code K08).

### Cancer Diagnosis

Topography and morphology codes were obtained from the Danish Cancer Registry^20^ and converted to *International Classification of Childhood Cancer, Third Edition* (*ICCC-3*) codes. The *ICCC-3* classification is based on pathology, whereas cancers in the *ICD* system are grouped according to anatomical location. To ease comparison across age groups, the *ICCC-3* classification was also used for adult-onset cancers. If a morphology code was missing (ie, no pathology sample available), an assigned *ICD-10* code in the registry was converted to a corresponding *ICCC-3* code. If assigning an *ICCC-3* code was not possible due to poor specification of the cancer type and location, the event was included only in the analysis of any cancer.

### Syndrome Diagnosis

A syndrome diagnosis was defined as either an abnormal karyotype (except balanced translocations) or abnormal chromosomal microarray registered in the Danish Cytogenetic Central Register^21^ or a syndrome *ICD* code in the Danish National Patient Registry, also including ectodermal dysplasia and incontinentia pigmenti, which are coded as malformations of the skin in the *ICD-10* (*ICD-8* codes 7592-7599 and *ICD-10* codes Q82.3-Q82.4 or within Q85-Q99) (detailed description in eMethods 2 in Supplement 1). Genetic testing for a syndrome registered in these data sources was performed as part of clinical care if the examining clinician suspected a syndromic diagnosis. Results of single-gene tests (eg, colorectal cancer panel screening, including *APC* and *AXIN2*) were not available. Tooth agenesis in itself was not an indication for genetic testing within the study period, and no testing was performed as part of this study.

### Statistical Analysis

Statistical analyses were performed from January through June 2023. Hazard ratios (HRs) and 95% CIs were estimated to evaluate associations between tooth agenesis and cancer. Survival time was calculated as days from date of birth until first diagnosis of cancer or censoring due to emigration, age of 40 years, death, or end of the study period, whichever came first. If cancer was diagnosed before birth, a risk time of 0.1 days was assigned. Models were evaluated by plots of observed and fitted survival curves and log-log survival curves. To meet the assumption of proportional hazards, the analyses were split into age groups (<1 year, 1 to <3 years, 3 to <10 years, 10 to <20 years, 20 to <30 years, and 30 to <40 years).^22,23^

We assessed HRs for any cancer and specific cancer types before 40 years of age. For each age group, analyses of specific cancer types were performed only if the number of cancer cases among the exposed was 5 or more. Associations with nonsyndromic tooth agenesis were evaluated by repeating the analyses after exclusion of individuals with a registered genetic syndrome, as defined above.

A sensitivity analysis was performed after further exclusion of cases in which detection of the tooth agenesis could be related to the clinical workup at the time of or after cancer diagnosis, possibly leading to detection bias. This exclusion was defined as cases in which the date of tooth agenesis diagnosis came after the date of cancer diagnosis and tooth agenesis was registered in the hospital system only (*ICD* codes obtained from the Danish National Patient Registry).

All analyses were performed using R, version 4.1.0, dplyr and survival packages (R Foundation for Statistical Computing). A detailed description of the statistical analyses is available as eMethods 3 in Supplement 1.

## Results

### Study Population

Among 2 501 715 included individuals (1 284 292 [51.3%] male and 1 217 423 [48.7%] female), 70 288 (2.8%) had a diagnosis of tooth agenesis and 26 308 (1.1%) had a diagnosis of early-onset cancer within the study period; 778 individuals had co-occurrence of tooth agenesis and cancer. The mean (SD) age at tooth agenesis diagnosis in our cohort was 13.2 (4.1) years, and the distribution of year of birth differed between individuals with and without tooth agenesis (Table). Among individuals with tooth agenesis, those who were registered in the hospital system only (12 019 [17.1%]) were more likely to be born before 1989 (4864 [40.5%] vs 8502 [14.6%]) and to have a syndrome diagnosis (359 [3.0%] vs 830 [1.4%]) compared with those with a tooth agenesis diagnosis in the municipal dental care system (eTable 1 in Supplement 1). Individuals with oligodontia (congenital absence of ≥6 teeth) had a higher occurrence of early-onset cancer (136 [1.2%]) compared with those with hypodontia (congenital absence of <6 teeth) (408 [0.9%]), but numbers were small (eTable 2 in Supplement 1).

**Table.** Cohort Characteristics by Tooth Agenesis^a^

Characteristic	Tooth agenesis		Total population (N = 2 501 715)
	Yes (n = 70 288)	No (n = 2 431 427)	
Sex			
Female	38 429 (54.7)	1 178 994 (48.5)	1 217 423 (48.7)
Male	31 859 (45.3)	1 252 433 (51.5)	1 284 292 (51.3)
Gestational age group, wk			
<32	406 (0.6)	14 858 (0.6)	15 264 (0.6)
32-36	2846 (4.0)	95 158 (3.9)	98 004 (3.9)
37-39	24 667 (35.1)	836 335 (34.4)	861 002 (34.4)
≥40	40 621 (57.8)	1 400 281 (57.6)	1 440 902 (57.6)
Missing	1748 (2.5)	84 795 (3.5)	86 543 (3.5)
Birth weight			
Mean (SD), g	3476 (566)	3477 (560)	3477 (560)
Missing	829 (1.2)	32 386 (1.3)	33 215 (1.3)
Birth year			
1977-1988	13 366 (19.0)	643 568 (26.5)	656 934 (26.3)
1989-1998	30 967 (44.1)	610 864 (25.1)	641 831 (25.7)
1999-2008	24 424 (34.7)	599 616 (24.7)	624 040 (24.9)
2009-2018	1531 (2.2)	577 379 (23.7)	578 910 (23.1)
Maternal age			
Mean (SD), y	28.8 (4.9)	28.8 (5.0)	28.8 (5.0)
Missing	829 (1.2)	32 386 (1.3)	33 215 (1.4)
Syndrome diagnosis			
Yes	1189 (1.7)	13 257 (0.5)	14 446 (0.6)
No	69 099 (98.3)	2 418 170 (99.5)	2 487 269 (99.4)
Cancer diagnosis			
Any	778 (1.11)	25 530 (1.05)	26 308 (1.08)
Multiple (≥2)	36 (0.05)	967 (0.04)	1003 (0.04)

^a^ Data are presented as number (percentage) unless otherwise indicated.

### Main Analyses

**Figure 3.**
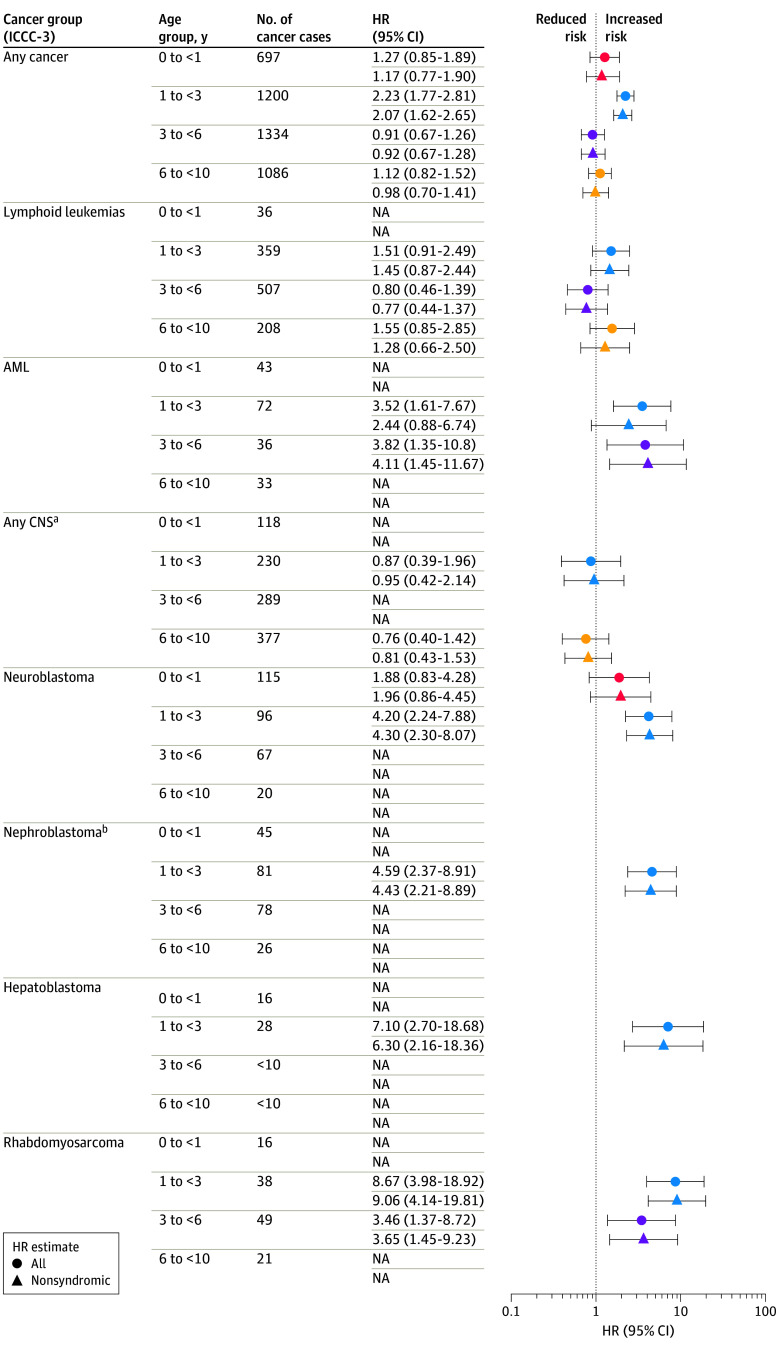
Hazard Ratios (HRs) and 95% CIs for Associations Between Congenital Tooth Agenesis and Any and Specific Cancers in Patients From Birth to Younger Than 10 Years AML indicates acute myeloid leukemia; CNS, central nervous system; ICCC-3, *International Classification of Childhood Cancer, Third Edition*; NA, not applicable (analysis was not performed because of the small number of individuals with both tooth agenesis and cancer [n < 5]). ^a^Central nervous system and miscellaneous intracranial and intraspinal neoplasms. ^b^Nephroblastoma and other nonepithelial kidney tumors.

Results of the main analyses are presented in Figure 3 (eTables 3-9 and eFigure in Supplement 1). Overall, tooth agenesis was associated with occurrence of any cancer in the 1 to younger than 3 years age group (HR, 2.23; 95% CI, 1.77-2.81) and the 30 to younger than 40 years age group (HR, 1.20; 95% CI, 1.02-1.40). In the analyses of specific cancers in childhood, we found associations with acute myeloid leukemia (AML) in the 1 to younger than 3 years age group (HR, 3.52; 95% CI, 1.61-7.67) and 3 to younger than 6 years age group (HR, 3.82; 95% CI, 1.35-10.8), neuroblastoma in the 1 to younger than 3 years age group (HR, 4.20; 95% CI, 2.24-7.88), nephroblastoma and other nonepithelial kidney tumors in the 1 to younger than 3 years age group (HR, 4.59; 95% CI, 2.37-8.91), hepatoblastoma in the 1 to younger than 3 years age group (HR, 7.10; 95% CI, 2.70-18.68), and rhabdomyosarcoma in the 1 to younger than 3 years (HR, 8.67; 95% CI, 3.98-18.92) and 3 to younger than 6 years age group (HR, 3.46; 95% CI, 1.37-8.72). Among adolescents and young adults, tooth agenesis was associated with osteosarcoma in the 10 to younger than 20 years age group (HR, 2.19; 95% CI, 1.11-4.32), carcinomas of the appendix in the 20 to younger than 30 years age group (HR, 2.54; 95% CI 1.03-6.24), carcinomas of colon and rectum in the 30 to younger than 40 years age group (HR, 2.81; 95% CI, 1.38-5.71), and carcinomas of the urinary bladder in the 20 to younger than 30 years age group (HR, 3.35; 95% CI, 1.35-8.30).

Associations with non-Hodgkin lymphoma and malignant melanoma were not consistent throughout age groups. In contrast with childhood-onset AML, we observed no association with AML among adolescents. Exclusion of individuals with a syndrome diagnosis had little effect on interpretation of the estimates, except for AML in the age group 1 to younger than 3 years; exclusion resulted in an attenuated HR of 2.44 (95% CI, 0.88-6.74).

### Sensitivity Analysis

Results of the sensitivity analysis are presented in eTables 3 to 9 in Supplement 1. After exclusion of co-occurrence cases in which identification of tooth agenesis could be related to detection bias, estimates were attenuated for most associations in childhood and adolescence (age <20 years). This finding was most pronounced for AML, non-Hodgkin lymphoma, and rhabdomyosarcoma, among which only the association with rhabdomyosarcoma in the age group 1 to younger than 3 years remained statistically significant (HR, 4.54; 95% CI, 1.60-12.92). For all other positive associations, estimates changed little or not at all.

## Discussion

In this population-based cohort study, tooth agenesis was associated with diagnosis of several cancer types before the age of 40 years. Overall, estimates changed little after exclusion of individuals with a known syndrome. We found an association with colorectal carcinomas among young adults, as previously suggested.^5^ This finding is in line with a previous association study that linked colorectal cancer–associated single nucleotide variants in the genes *ATF1* (OMIM 123803), *DUSP10* (OMIM 608867), and *CASC8* (OMIM 617701) with clinically verified tooth agenesis.^9^ In contrast, a case-control study using data from a colorectal cancer family registry found that self-reported tooth agenesis occurred with the same frequency among patients with colorectal cancer and relatives without colorectal cancer.^24^ However, the use of family members as controls may hinder detection of an association if familial genetic variants cause both tooth agenesis with high penetrance and colorectal cancer with low penetrance and later onset.

Among young adults, we also observed associations with carcinomas of the appendix (a rare cancer type that is often a low-grade incidental finding in young individuals undergoing appendectomy^25,26^) and carcinomas of the bladder. These associations with adult-onset carcinomas could be unrelated or could reflect one shared genetic predisposition (either a novel cancer predisposition syndrome or a broader phenotype of an established syndrome). A shared genetic cause is not implausible because colorectal and bladder cancers are known to co-occur in *MSH2* (OMIM 609309)–related Lynch syndrome,^27^ and carcinomas of the appendix are associated with occurrence of other malignant tumors of the gastrointestinal tract.^25,26^

In embryonic development, teeth, skin, and melanocytes are of ectodermal origin, which could be part of the underlying mechanism of the apparent association with malignant melanoma. However, in analyses restricted to nonsyndromic cases, also excluding ectodermal dysplasia and related conditions, the estimates did not change and thus did not point toward these conditions as part of an underlying mechanism. Because associations were not consistent throughout age groups, this may be a chance finding.

The negative association with cervical cancer in young adults may also be a chance finding or could be due to social or other nongenetic factors. For example, it is possible that factors associated with the risk of human papillomavirus infection could be more or less frequent among individuals with tooth agenesis.^28^ Ultimately, although notable, this finding requires additional investigation.

Due to the large size of our study, we were also able to assess associations with rare cancer types in childhood and adolescence. We observed several novel associations that should be interpreted with caution, as the risk of bias is influenced by the natural timing of tooth eruption for these age groups. However, several associations are supported with established biological pathways. For example, the observed association with hepatoblastoma is supported by the well-established link between *APC*-related familial adenomatous polyposis and hepatoblastoma^29^ and between familial adenomatous polyposis and tooth anomalies.^30^ We also observed associations with other extracranial embryonal tumors in childhood (eg, neuroblastoma and nephroblastoma), and estimates for these cancer types changed little in our sensitivity analysis; this consistency further supports the validity of our findings. The Wnt pathway, including somatic *APC* mutations, is also known to be implicated in the development of osteosarcomas,^31,32^ for which we found an association with tooth agenesis in adolescence.

Overall, associations between tooth agenesis and AML were not consistent. Specifically, in the 1 to younger than 3 years age group, results were attenuated in the analysis restricted to nonsyndromic cases, which could be due to exclusion of individuals with Down syndrome. For both this and the 3 to younger than 6 years age group, estimates were attenuated in the sensitivity analysis and failed to remain statistically significant. In AML, treatment with chemotherapy is extensive and hematopoietic stem cell transplantation is often required. Because these treatments are reported to cause aplasia of the permanent dentition in young children,^33^ which may later be misdiagnosed as tooth agenesis, it has to be considered that the apparent association with AML could be treatment related rather than congenital.

As with AML, the association with rhabdomyosarcoma was affected greatly in the sensitivity analysis. Because rhabdomyosarcoma presents around the head and neck in approximately 40% of cases,^34^ it is possible that tooth agenesis is more likely to be incidentally detected in children with this cancer type and that the apparent association is in fact, or at least partially, due to detection bias.

We hypothesize that the co-occurrence of cancers and tooth agenesis in our study could have a shared genetic cause, possibly within the Wnt pathway. The associations with childhood- and adult-onset cancers may represent different genetic entities. Genetic evaluation of the associations could clarify the possible clinical implications, in particular, the rationale for genetic testing of individuals with tooth agenesis, cancer surveillance, and possibility of future targeted cancer treatments.

### Strengths and Limitations

Our study has several strengths. The Danish national registries contain prospectively collected data of high quality and completeness.^35^ In particular, the Danish Central Registry of Odontology represents a unique opportunity to investigate tooth agenesis in a large-scale setting, as it is based on professional clinical examinations and has little selection bias because more than 95% of Danish children participate in the program.^36^

Our study also has some limitations. Although a register-based approach has the strength of providing a large number of observations, it provides no proof of causation, and the design of the study is likely to introduce bias. The natural timing of tooth eruption represents a particular challenge in a longitudinal study of tooth agenesis because the condition is congenital but does not present at birth. The mean (SD) age at tooth agenesis diagnosis in our cohort was 13.2 (4.1) years, which was also reflected in that very few of those who were born by the end of the study period had received a tooth agenesis diagnosis (Table). This misclassification caused by diagnostic delay could bias the estimates of associations in childhood toward or away from the null, depending on whether the delay is shorter in patients with childhood cancer.

Indeed, there is a risk of detection bias influencing the results if being diagnosed with cancer increases the probability of detection of tooth agenesis. For some years, a full dental workup was performed at the time of cancer diagnosis in young children at some Danish pediatric cancer institutions, and tooth agenesis detected at this examination would be registered in the Danish National Patient Registry. We attempted to account for this possible detection bias in a sensitivity analysis and found that estimates were attenuated for many associations in childhood and adolescence (age <20 years).

As mentioned earlier, another challenge in interpretation of the results among children younger than 6 years is that early exposure to chemotherapy and hematopoietic stem cell transplantation is reported to cause aplasia of the permanent dentition,^33^ which could later be miscoded as congenital absence of teeth, leading to reverse causation. However, studies^33,37^ on dental late effects only include examinations after treatment, and thus some of the dental anomalies reported to be treatment related could in fact be congenital. If possible, future studies of the tooth agenesis and cancer association should include pretreatment dental examinations to overcome these challenges.

Another drawback of the prospective cohort design is the need for long follow-up time. In adults, an association between tooth agenesis and ovarian cancer has been suggested,^6,7,38^ but we were not able to evaluate this possible association in our study due to very few cases before 40 years of age within the study period. Indeed, being able to extend the study period to include cancers until 50 years of age may have disclosed further and/or stronger associations, as the increased cancer risk with genetic predisposition is not limited to very early-onset cases. Accordingly, in *AXIN2*-related oligodontia–colorectal cancer syndrome, colorectal cancers are typically diagnosed after the age of 40 years, although polyps can present in adolescence.^12,39,40,41^

Finally, multiple comparisons were made, all of which are presented in eTables 3 to 9 in Supplement 1 (across age groups and tumor types: 50 main analyses with 50 subanalyses excluding syndromic cases and 15 sensitivity analyses). Because we did not account for this in the statistical analyses, some chance findings are to be expected as suggested above, and interpretations should be made accordingly.

## Conclusions

This cohort study supports previous evidence suggesting an association between tooth agenesis and colorectal cancer risk in early adulthood. Our findings also suggest associations with several other specific cancer types, in particular extracranial embryonal tumors in childhood. However, the absolute number of tooth agenesis–cancer co-occurrences within the study period was low. Improved understanding of the genetic causes of the specific associations is needed to assess possible clinical implications.

## Notes

Supplement 1. **eMethods 1.** Detailed Description of Data Sources in the Danish Health Care System **eMethods 2.** Detailed Description of Syndrome Diagnosis **eMethods 3.** Detailed Description of Statistical Analyses **eTable 1.** Tooth Agenesis Population Characteristics Stratified on Registry **eTable 2.** Danish Central Registry of Odontology Subpopulation Characteristics Stratified on Tooth Agenesis Severity **eTable 3.** Results by Age Groups, 0 to <1 Years **eTable 4.** Results by Age Groups, 1 to <3 Years **eTable 5.** Results by Age Groups, 3 to <6 Years **eTable 6.** Results by Age Groups, 6 to <10 Years **eTable 7.** Results by Age Groups, 10 to <20 Years **eTable 8.** Results by Age Groups, 20 to <30 Years **eTable 9.** Results by Age Groups, 30 to <40 Years **eFigure.** Hazard Ratio (HRs) and 95% CIs for Associations Between Congenital Tooth Agenesis and Any and Specific Cancers in Patients Aged 10 to Younger Than 40 Years

Supplement 2.
Data Sharing Statement
