# Supplementary material for: Congenital Tooth Agenesis and Risk of Early-Onset Cancer
Source: JAMA Netw Open. 2024 Mar 15;7(3):e240365. doi: 10.1001/jamanetworkopen.2024.0365 (PMC10943407; doi:10.1001/jamanetworkopen.2024.0365)
Supplement: Supplement 1. — eMethods 1. Detailed Description of Data Sources in the Danish Health Care System eMethods 2. Detailed Description of Syndrome Diagnosis eMethods 3. Detailed Description of Statistical Analyses eTable 1. Tooth Agenesis Population Characteristics Stratified on Registry eTable 2. Danish Central Registry of Odontology Subpopulation Characteristics Stratified on Tooth Agenesis Severity eTable 3. Results by Age Groups, 0 to <1 Years eTable 4. Results by Age Groups, 1 to <3 Years eTable 5. Results by Age Groups, 3 to <6 Years eTable 6. Results by Age Groups, 6 to <10 Years eTable 7. Results by Age Groups, 10 to <20 Years eTable 8. Results by Age Groups, 20 to <30 Years eTable 9. Results by Age Groups, 30 to <40 Years eFigure. Hazard Ratio (HRs) and 95% CIs for Associations Between Congenital Tooth Agenesis and Any and Specific Cancers in Patients Aged 10 to Younger Than 40 Years [file jamanetwopen-e240365-s001.pdf]

Supplementary Online Content

Eiset SE, Schraw J, Sørensen GV, et al. Congenital tooth agenesis and risk of early-onset cancer. *JAMA Netw Open*. 2024;7(3):e240365. doi:10.1001/jamanetworkopen.2024.0365

eMethods 1. Detailed Description of Data Sources in the Danish Health Care System

eMethods 2. Detailed Description of Syndrome Diagnosis

eMethods 3. Detailed Description of Statistical Analyses

eTable 1. Tooth Agenesis Population Characteristics Stratified on Registry

eTable 2. Danish Central Registry of Odontology Subpopulation Characteristics Stratified on Tooth Agenesis Severity

eTable 3. Results by Age Groups, 0 to <1 Years

eTable 4. Results by Age Groups, 1 to <3 Years

eTable 5. Results by Age Groups, 3 to <6 Years

eTable 6. Results by Age Groups, 6 to <10 Years

eTable 7. Results by Age Groups, 10 to <20 Years

eTable 8. Results by Age Groups, 20 to <30 Years

eTable 9. Results by Age Groups, 30 to <40 Years

eFigure. Hazard Ratio (HRs) and 95% CIs for Associations Between Congenital Tooth Agenesis and Any and Specific Cancers in Patients Aged 10 to Younger Than 40 Years

This supplementary material has been provided by the authors to give readers additional information about their work.

© 2024 Eiset SE et al. *JAMA Network Open.*

eMethods 1: Detailed description of data sources in the Danish health care system

The Danish health care system has been almost entirely tax funded since 1970, and includes free access to general practitioners, outpatient clinics, and hospitals (1). While socioeconomic factors are still an important factor for individual health, even in a free-of-charge and easily accessible setting, a Danish study of socioeconomics in relation to diagnosis of pediatric cancer showed no association between socioeconomic position and stage of disease at diagnosis (2).
Danish health care data is routinely collected in several databases, and linkage is possible through the unique 10-digit Civil Registration number assigned to all Danish residents (3). The registries linked in our study are described below.

The Danish Civil Registration System (4)
The Danish Civil Registration system was established in 1968. The registry includes information on migration and vital status of Danish residents, which can be linked to other registries by unique individual 10-digit Civil Registration numbers.

The Danish National Patient Registry (5)
The Danish National Patient Registry contains discharge information from Danish non-psychiatric hospitals from 1977, with complete nationwide coverage from 1978. Outpatient hospital encounters, specialty clinics and emergency rooms are included from 1994, and complete from 1995. For each encounter, one primary and optional secondary diagnoses are recorded.
Diagnoses have been registered according to Danish adaptions of the eighth revision of the International Classification of Diseases (ICD-8) through 1993, and the tenth edition (ICD-10) from 1994 onwards. The Danish adaption of ICD-8 was modified to include two extra digits and is to some extent comparable to the international ICD-9 classification, which was never introduced in Denmark.
Tooth agenesis diagnosis is registered as ICD-8 code 520.09 and ICD-10 K00.0, both denoted anodontia (complete congenital absence of all deciduous and permanent teeth), but also applicable to partial anodontia. In ICD-10, K00.0 subcodes for hypodontia (congenital lack of 1-5 permanent teeth, third molars excluded) and oligodontia (congenital lack of 6 or more permanent teeth, third molars excluded) were available until 2012. A third subcode for tooth aplasia is now being used clinically for cases of hypodontia, although aplasia in biological terms is not completely synonymous to agenesis. All codes are applicable to congenital absence of teeth only, thus excluding acquired absence of teeth.

The Danish Central Registry of Odontology (6, 7, 8)
The Danish Central Registry of Odontology has collected data from the Danish municipal dental care since 1972. In Denmark, dental care has been free of charge and easily accessible for children and adolescents younger than 18 years from 1972 through 2021, and was extended to include individuals younger than 22 years from 2022. Danish children are automatically signed up for the program at birth and >95% of Danish children participate (4).
Registrations were made annually from 1972 through 1992. From 1993, registrations have only been mandatory for children aged 5, 7, 12, or 15 years, but are still possible at other ages. Registration of tooth agenesis (hypodontia and oligodontia) began in 1988.

The Danish Cancer Registry (9)
The Danish Cancer Registry contains data on incident malignant neoplasms in Denmark since 1943, with mandatory registration since 1987. Tumor characteristics in the registry include ICD-O-3 classification morphology codes (available for 89% of registered tumors) and topography codes, and ICD-10 cancer codes based on clinical and histological evaluation. The registry contains information on multiple primary tumors, but not within the same organ.

The Danish Cytogenetic Central Register (10, 11)
The Danish Cytogenetic Central Register has collected data on germline karyotypes performed in Denmark since 1960, and contains all pre- and postnatal karyotypes and chromosomal microarray test results in the study period.
Antenatal care is free for all pregnant Danish residents and includes 1st and 2nd trimester ultrasonic scans (12). Prenatal screening for aneuploidies by combined test in the first trimester has been offered to all pregnant women in Denmark since 2004 (13). Since 2013, chromosomal microarray has been the standard analysis offered to the parents of high-risk pregnancies (14). Before 2004, prenatal karyotyping was performed at maternal age above 35 years or by maternal request.
Postnatal genetic testing for a syndrome were performed as part of clinical care if the examining clinician suspected a syndromic diagnosis. Tooth agenesis in itself was not an indication for genetic testing within the study period.

References

(1) Christiansen T. Organization and financing of the Danish health care system. Health Policy. 2002;59(2):107-118. doi:10.1016/s0168-8510(01)00201-9

(2) Pedersen LH, Erdmann F, Aalborg GL, et al. Socioeconomic position and prediagnostic health care contacts in children with cancer in Denmark: a nationwide register study. BMC Cancer. 2021;21(1):1104. Published 2021 Oct 14. doi:10.1186/s12885-021-08837-x

© 2024 Eiset SE et al. *JAMA Network Open.*

(3) Schmidt M, Schmidt SAJ, Adelborg K, et al. The Danish health care system and epidemiological research: from health care contacts to database records. Clin Epidemiol. 2019;11:563-591. Published 2019 Jul 12. doi:10.2147/CLEP.S179083

(4) Pedersen LH, Erdmann F, Aalborg GL, et al. Socioeconomic position and prediagnostic health care contacts in children with cancer in Denmark: a nationwide register study. BMC Cancer. 2021;21(1):1104. Published 2021 Oct 14. doi:10.1186/s12885-021-08837-x

(5) Schmidt M, Schmidt SA, Sandegaard JL, Ehrenstein V, Pedersen L, Sørensen HT. The Danish National Patient Registry: a review of content, data quality, and research potential. Clin Epidemiol. 2015;7:449-490. Published 2015 Nov 17. doi:10.2147/CLEP.S91125

(6) https://sundhedsdatastyrelsen.dk/da/registre-og-services/om-de-nationale-sundhedsregistre/graviditet-foedsler-og-boern/boern_ungdom_tandpleje (accessed June 28 2023)

(7) https://www.sst.dk/da/Viden/Sundhedsvaesen/Det-primaere-sundhedsvaesen/Tandsundhed/Boerne--og-ungdomstandpleje (accessed June 28 2023)

(8) Nørrisgaard PE, Qvist V, Ekstrand K. Prevalence, risk surfaces and inter-municipality variations in caries experience in Danish children and adolescents in 2012. Acta Odontol Scand. 2016;74(4):291-297. doi:10.3109/00016357.2015.1119306

(9) Gjerstorff ML. The Danish Cancer Registry. Scand J Public Health. 2011;39(7 Suppl):42-45. doi:10.1177/1403494810393562

(10) Videbech, P. and J. Nielsen, Electronic data processing in the Danish cytogenetic central register and EDP problems of registers in general. Clin Genet, 1979. 15(2): p. 137-46.

(11) https://www.fagperson.auh.dk/afdelinger/klinisk-genetisk-afdeling/dccr/om-dccr/ (accessed June 28 2023)

(12) https://www.sst.dk/-/media/Udgivelser/2022/Ukraine/Gravid/Ukraine-gravid-EN.ashx (accessed November 17, 2023)

(12) Ekelund CK, Jørgensen FS, Petersen OB, Sundberg K, Tabor A; Danish Fetal Medicine Research Group. Impact of a new national screening policy for Down's syndrome in Denmark: population based cohort study. BMJ. 2008;337:a2547. Published 2008 Nov 27. doi:10.1136/bmj.a2547

(13) https://www.sst.dk/-/media/Udgivelser/2020/Fosterdiagnostik/Retningslinjer-for-fosterdiagnostik.ashx (accessed November 17, 2023)

(14) Nørgaard LN, Ekelund C, Fagerberg C, et al. Array-komparativ genomisk hybridisering er en ny og lovende metode til prænatal kromosomundersøgelse [Array-comparative genomic hybridization is a new and promising method for prenatal chromosomal diagnosis]. Ugeskr Laeger. 2014;176(30):1379-1382.

© 2024 Eiset SE et al. *JAMA Network Open.*

eMethods 2: Detailed description of syndrome diagnosis

Two sources were used for identification of a known syndrome: The Danish Cytogenetic Central Register (1, 2) and the Danish National Patient Registry (3).

All individuals with an abnormal karyotype (except balanced translocations) or abnormal chromosomal microarray in the Danish Cytogenetic Central Register (described in eMethods1) were classified as having a syndrome diagnosis. Tooth agenesis in itself was not an indication for genetic screening within the study period.

We were not able to include information about single-gene tests and whole exome/whole genome sequencing, but instead used diagnoses by International Classification of Diseases (ICD) codes for further identification of syndromes. From the Danish National Patient Registry, we classified individuals as having a syndrome diagnosis, if they had been assigned any of the following ICD codes:

ICD-8 (1977 through 1993)

75929: Other or unspecified deformities

75931 Down Syndrome, translocation t(13:21)

75939 Down Syndrome

75940 Trisomy 18

75941 Trisomy 13

75942 Cri du chat

75949 Other significant anomaly of autosomes

75950 Turner Syndrome

75951 Klinefelter Syndrome

75958 Other specified anomaly of sex chromosomes

75959 Anomaly of sex chromosomes, unspecified

75969 Tuberous sclerosis

75980 Marfan Syndrome

75981 Bardet-Biedl Syndrome

75982 Sturge-Weber Syndrome

75983 Ataxia Telangiectasia

75989 Other specified syndrome with multiple congenital malformations

75999 Multiple congenital malformations, unspecified

ICD-10 (1994 onwards)

Q82.3 Incontinentia pigmenti (with subcodes)

Q82.4 Ectodermal dysplasia (with subcodes)

Q85 Phakomatoses, not elsewhere classified

Q85.0 Neurofibromatosis von Recklinghausen

Q85.1 Tuberous sclerosis

Q85.8 Other phakomatoses

Q85.8A Kartagener's Syndrome (*1994 through 2011*)

Q85.8B Peutz-Jegher Syndrome

Q85.8C Sturge-Weber Syndrome

Q85.8D Von Hippel-Lindau Syndrome

Q87 Other specified congenital malformation syndromes affecting multiple systems

Q87.0 Congenital malformation syndromes predominantly affecting facial appearance

Q87.0A Acrocephalopolysyndactylia

Q87.0B Apert syndrome

Q87.0C Cryptophthalmos

Q87.0D Cyclopia

Q87.0E Franceschetti syndrome (*1994 through 2011*)

Q87.0F Goldenhar-Gorlin syndrome

Q87.0G Moebius Syndrome

Q87.0H Oral-Facial-Digital syndrome

Q87.0I Pierre Robins syndrome

Q87.0J Robin syndrome (*1994 through 2011*)

Q87.0K Synophthalmia (*1994 through 2011*)

Q87.0L Treacher-Collins syndrome (*1994 through 2011*)

Q87.0M Waardenburg syndrome

Q87.1 Congenital malformation syndromes predominantly associated with short stature

Q87.1A Cockayne syndrome

Q87.1B Cornelia de Langes syndrome

Q87.1C Dubowitz syndrome

Q87.1D Noonan syndrome

Q87.1E Prader-Willi syndrome

Q87.1F Robinow syndrome

Q87.1G Silver-Russell syndrome

Q87.1H Seckel syndrome

Q87.1I Smith-Lemli-Opitz syndrome

Q87.1J Faciogenital dysplasia (Aarskog-Scott syndrome)

Q87.2 Congenital malformation syndromes predominantly involving limbs

Q87.2A Holt-Oram syndrome

Q87.2B Klippel-Trenaunay-Weber syndrome

© 2024 Eiset SE et al. *JAMA Network Open.*

Q87.2C Nail-patella-syndrome

Q87.2D Rubinstein-Taybi syndrome

Q87.2E Sirenomelia

Q87.2F Thrombocytopenia with aplasia radii (TAR)

Q87.2G VATER syndrome

Q87.3 Congenital malformation syndromes involving early overgrowth

Q87.3A Beckwith-Wiedemann syndrome

Q87.3B Sotos syndrome

Q87.3C Weaver syndrome

Q87.4 Marfan syndrome

Q87.4A Arachnodactylia

Q87.5 Other congenital malformation syndromes with other skeletal changes

Q87.8 Other specified congenital malformation syndromes, not elsewhere classified

Q87.8A Alport syndrome

Q87.8B Laurence-Moon-Biedl-Bardet syndrome

Q87.8C Zellweger syndrome

Q87.8D Cowden syndrom

Q87.8E Goltz syndrom

Q87.8F Rothmund-Thomson syndrome

Q87.8G Haber syndrom (*1994 through 2011*)

Q87.8H Keratosis palmoplantaris with parodontitis

Q87.8I Poikiloderma congenitale (Rothmund-Thomson)

Q87.8J LEOPARD syndrome

Q87.8K Birt-Hogg-Dube syndrom

Q90 Down syndrome (*with subcodes*)

Q91 Edwards syndrome and Patau syndrome (*with subcodes*)

Q92 Other trisomies and partial trisomies of the autosomes, not elsewhere classified (*with subcodes*)

Q93 Monosomies and deletions from the autosomes, not elsewhere classified

Q93.0 Whole chromosome monosomy, nonmosaicism (meiotic nondisjunction)

Q93.1 Whole chromosome monosomy, mosaicism (mitotic nondisjunction)

Q93.2 Chromosome replaced with ring, dicentric or isochromosome (*with subcodes*)

Q93.3 Deletion of short arm of chromosome 4

Q93.3A Wolff-Hirschorn syndrome (*1994 through 2011*)

Q93.4 Deletion of short arm of chromosome 5

© 2024 Eiset SE et al. *JAMA Network Open.*

Q93.4A Cri-du-chat syndrom (*1994 through 2011*)

Q93.5 Other deletions of part of a chromosome

Q93.5A Lejeune syndrome (*1994 through 2011*)

Q93.5B Partiel translokation 4-5

Q93.5C Angelman syndrome

Q93.6 Deletions only visible in prometaphase

Q93.7 Deletions with other complex rearrangements

Q93.8 Other deletions from the autosomes

Q93.9 Deletion from autosomes, unspecified

Q96 Turner syndrome (with subcodes)

Q97 Other sex chromosome abnormalities, female phenotype, not elsewhere classified

Q97.0 Karyotype 47,XXX

Q97.1 Female with more than three X chromosomes

Q97.2 Mosaicism, lines with various numbers of X chromosomes

Q97.3 Female with 46, XY karyotype

Q97.8 Other specified sex chromosome abnormalities, female phenotype

Q97.9 Sex chromosome abnormality, female phenotype, unspecified

Q98 Other sex chromosome abnormalities, male phenotype, not elsewhere classified

Q98.0 Klinefelter syndrome karyotype 47, XXY

Q98.1 Klinefelter syndrome, male with more than two X chromosomes

Q98.2 Klinefelter syndrome, male with karyotype 46,XX

Q98.3 Male with karyotype 46,XX without Klinefelter syndrome

Q98.4 Klinefelter syndrome, unspecified

Q98.5 Karyotype 47, XYY

Q98.6 Male with structurally abnormal sex chromosome

Q98.7 Male with sex chromosome mosaicism

Q98.8 Other specified sex chromosome abnormalities, male phenotype (with subcodes)

Q98.9 Sex chromosome abnormality, male phenotype, unspecified

Q99 Other chromosome abnormalities, not elsewhere classified

Q99.0 Chimera 46, XX/46, XY

Q99.1 46, XX true hermaphrodite (with subcodes)

Q99.2 Fragile X chromosome

Q99.8 Other specified chromosome abnormalities

Q99.9 Chromosomal abnormality, unspecified

References:

(1) Videbech, P. and J. Nielsen, Electronic data processing in the Danish cytogenetic central register and EDP problems of registers in general. Clin Genet, 1979. 15(2): p. 137-46.

(2) https://www.fagperson.auh.dk/afdelinger/klinisk-genetisk-afdeling/dccr/om-dccr/ (accessed June 28 2023)

(3) Schmidt M, Schmidt SA, Sandegaard JL, Ehrenstein V, Pedersen L, Sørensen HT. The Danish National Patient Registry: a review of content, data quality, and research potential. Clin Epidemiol. 2015;7:449-490. Published 2015 Nov 17. doi:10.2147/CLEP.S91125

© 2024 Eiset SE et al. *JAMA Network Open.*

eMethods 3: Detailed description of statistical analyses

Survival time was calculated as days from date of birth until first diagnosis of cancer (registered in the Danish Cancer Registry) or censoring due to emigration, age 40 years, death, or end of the study period (registered in the Danish Civil Registration Registry), whichever came first. If cancer was diagnosed before birth, a risk time of 0.1 days was assigned.

Hazard ratios (HRs) and 95% confidence intervals (CIs) were estimated to evaluate associations between tooth agenesis and each cancer type. Evaluation of the models were made by examination of plots of observed and fitted survival curves, and log-log survival curves, and showed non-proportionality.

To meet the assumption of proportional hazards, the analyses were split into age groups (<1 year, 1 to <3 years, 3 to <10 years, 10 to <20 years, 20 to <30 years and 30 to <40 years) (1, 2). This approach was chosen as the age groups also accommodate the natural biology of the outcome in question – specific cancers occur at specific age windows. All individuals were right censored as described above, e.g., an individual with a survival time of 1500 days would contribute survival time to the analyses of the <1 year, 1 to <3 years, and 3 to <10 years age groups.

We assessed HRs for any cancer and specific cancer types before age 40 years. For each age group, analyses of specific cancer types were performed only if the number of cancer cases among the exposed was ≥5, in accordance with the Danish Health Data regulations.

Associations with non-syndromic tooth agenesis were evaluated by repeating the analyses after exclusion of individuals with a registered genetic syndrome, as defined in eMethods 2.

A sensitivity analysis was performed after further exclusion of cases where detection of the tooth agenesis could be related to the clinical workup at the time of or after cancer diagnosis, possibly leading to detection bias. This was defined as cases where

1. the date of tooth agenesis diagnosis came after the date of cancer diagnosis, and
2. tooth agenesis was registered in the hospital system only (ICD codes obtained from the Danish National Patient Registry)

as this could be influenced by having a cancer diagnosis. This is in contrast to the municipal dental care system, were nearly all Danish children are evaluated at least yearly from age two years.

All analyses were performed using R, version 4.1.0, *dplyr* and *survival* packages.

References:

(1) Zhang, Z., et al., Time-varying covariates and coefficients in Cox regression models. Ann Transl Med, 2018. 6(7): p. 121.
(2) Therneau et al. 2023, https://cran.r-project.org/web/packages/survival/vignettes/timedep.pdf

© 2024 Eiset SE et al. *JAMA Network Open.*

eTable 1. Tooth agenesis population characteristics stratified on registry

	Tooth agenesis data source		
	Danish Central Registry of Odontology<br><br>n = 58,269 (82.9%)	Danish National Patient Registry only<br><br>n = 12,019 (17.1%)	Overall<br><br>n = 70,288
**Characteristics**			
Age at diagnosis in years, mean (SD)	12.5 (3.4)	16.8 (4.9)	13.2 (4.1)
Birth year, n (%)			
1977-1988	8,502 (14.6%)	4,864 (40.5%)	13,366 (19.0%)
1989-1998	26,146 (44.9%)	4,821 (40.1%)	30,967 (44.1%)
1999-2008	22,159 (38.0%)	2,265 (18.8%)	24,424 (34.7%)
2009-2018	1,462 (2.5%)	69 (0.6%)	1,531 (2.2%)
Syndrome diagnosis, n (%)			
Yes	830 (1.4%)	359 (3.0%)	1,189 (1.7%)
No	57,439 (98.6%)	11,660 (97.0%)	69,099 (98.3%)

The left tooth agenesis column includes those registered in the Danish Central Registry of Odontology (municipal dental care), with or without additional registration in the Danish National Patient Registry (specialized hospital setting).

The right tooth agenesis column includes those registered in the Danish National Patient Registry without having been registered in the Danish Central Registry of Odontology.

© 2024 Eiset SE et al. *JAMA Network Open.*

eTable 2. Danish Central Registry of Odontology subpopulation characteristics stratified on tooth agenesis severity

	**Hypodontia**<br>**n = 46,822 (80.4%)**	**Oligodontia**<br>**n = 11,447 (19.6%)**	**Total**<br>**n = 58,269**
**Characteristics**			
Sex, n (%)			
Female	25,991 (55.5%)	5,741 (50.2%)	31,732 (54.5%)
Male	20,831 (44.5%)	5,706 (49.8%)	26,537 (45.5%)
Syndrome diagnosis, n (%)			
Yes	627 (1.34%)	175 (1.53%)	820 (1.41%)
No	46,195 (98.66%)	11,272 (98.47%)	57,449 (98.59%)
Cancer diagnosis, n (%)			
Any cancer before age 40 years	408 (0.87%)	136 (1.19%)	544 (0.93%)

Individuals registered in the Danish Central Registry of Odontology (municipal dental care), with or without additional registration in the Danish National Patient Registry (specialized hospital setting) (82.9% of tooth agenesis cases, see eTable 1)

Hypodontia is defined as congenital absence of <6 teeth (not including 3rd molars)

Oligodontia is defined as congenital absence of 6 teeth or more (not including 3rd molars). This includes anodontia (congenital absence of all teeth)

© 2024 Eiset SE et al. *JAMA Network Open.*

eTable 3. Results by age groups, 0 to <1 years: HR and 95% CI for overall, non-syndromic and sensitivity analyses

		Analysis estimates, HR (95% CI)		
	n	Tooth agenesis	Non-syndromic tooth agenesis	Sensitivity analysis
**Any cancer**	697	1.27 (0.85; 1.89)	1.17 (0.77; 1.90)	-
**Neuroblastoma**	115	1.88 (0.83; 4.28)	1.96 (0.86; 4.45)	1.63 (0.67; 4.00)

Non-syndromic tooth agenesis: Re-analysis after exclusion of individuals with known genetic syndromes

Sensitivity analysis: Re-analysis after further exclusion of cases where the date of tooth agenesis diagnosis came after the date of cancer diagnosis, and tooth agenesis was registered in the hospital system only (ICD codes obtained from the Danish National Patient Registry)

Abbreviations: HR, hazard ratio; CI, confidence interval; n, number of cancer cases in age group

eTable 4. Results by age groups, 1 to <3 years: HR and 95% CI for overall, non-syndromic and sensitivity analyses

		Analysis estimates, HR (95% CI)		
	n	Tooth agenesis	Non-syndromic tooth agenesis	Sensitivity analysis
**Any cancer**	1,200	2.23 (1.77; 2.81)	2.07 (1.62; 2.65)	-
**Lymphoid leukemias**	359	1.51 (0.91; 2.49)	1.45 (0.87; 2.44)	-
**AML**	72	3.52 (1.61; 7.67)	2.44 (0.88; 6.74)	1.84 (0.57; 5.87)
**Any cancer of the CNS**[a]	230	0.87 (0.39; 1.96)	0.95 (0.42; 2.14)	-
**Neuroblastoma**	96	4.20 (2.24; 7.88)	4.30 (2.30; 8.07)	3.13 (1.52; 6.47)
**Nephroblastoma**[b]	81	4.59 (2.37; 8.91)	4.43 (2.21; 8.89)	3.94 (1.89; 8.21)
**Hepatoblastoma**	28	7.10 (2.70; 18.68)	6.30 (2.16; 18.36)	6.30 (2.16; 18.36)
**Rhabdomyosarcomas**	38	8.67 (3.98; 18.92)	9.06 (4.14; 19.81)	4.54 (1.60; 12.92)

Non-syndromic tooth agenesis: Re-analysis after exclusion of individuals with known genetic syndromes

Sensitivity analysis: Re-analysis after further exclusion of cases where the date of tooth agenesis diagnosis came after the date of cancer diagnosis, and tooth agenesis was registered in the hospital system only (ICD codes obtained from the Danish National Patient Registry)

Abbreviations: HR, hazard ratio; CI, confidence interval; n, number of cancer cases in age group; AML, acute myeloid leukemia; CNS, central nervous system

[a]Including intracranial and intraspinal tumors

[b] and other nonepithelial renal tumors

© 2024 Eiset SE et al. *JAMA Network Open.*

eTable 5. Results by age groups, 3 to <6 years: HR and 95% CI for overall, non-syndromic and sensitivity analyses

		Analysis estimates, HR (95% CI)		
	n	Tooth agenesis	Non-syndromic tooth agenesis	Sensitivity analysis
**Any cancer**	1,334	0.91 (0.67; 1.26)	0.92 (0.67; 1.28)	-
**Lymphoid leukemias**	507	0.80 (0.46; 1.39)	0.77 (0.44; 1.37)	-
**AML**	36	3.82 (1.35; 10.80)	4.11 (1.45; 11.67)	3.09 (0.94; 10.13)
**Rhabdomyosarcomas**	49	3.46 (1.37; 8.72)	3.65 (1.45; 9.23)	0.73 (0.10; 5.32)

Non-syndromic tooth agenesis: Re-analysis after exclusion of individuals with known genetic syndromes

Sensitivity analysis: Re-analysis after further exclusion of cases where the date of tooth agenesis diagnosis came after the date of cancer diagnosis, and tooth agenesis was registered in the hospital system only (ICD codes obtained from the Danish National Patient Registry)

Abbreviations: HR, hazard ratio; CI, confidence interval; n, number of cancer cases in age group; AML, acute myeloid leukemia

© 2024 Eiset SE et al. *JAMA Network Open.*

eTable 6. Results by age groups, 6 to <10 years: HR and 95% CI for overall, non-syndromic and sensitivity analyses

		Analysis estimates, HR (95% CI)		
	n	Tooth agenesis	Non-syndromic tooth agenesis	Sensitivity analysis
**Any cancer**	1,086	1.12 (0.82; 1.52)	0.98 (0.70; 1.38)	-
**Lymphoid leukemias**	208	1.55 (0.85; 2.85)	1.28 (0.66; 2.50)	-
**Any cancer of the CNS[a]**	377	0.76 (0.40; 1.42)	0.81 (0.43; 1.53)	-

Non-syndromic tooth agenesis: Re-analysis after exclusion of individuals with known genetic syndromes

Sensitivity analysis: Re-analysis after further exclusion of cases where the date of tooth agenesis diagnosis came after the date of cancer diagnosis, and tooth agenesis was registered in the hospital system only (ICD codes obtained from the Danish National Patient Registry)

Abbreviations: HR, hazard ratio; CI, confidence interval; n, number of cancer cases in age group; CNS, central nervous system

[a]Including intracranial and intraspinal tumors

© 2024 Eiset SE et al. *JAMA Network Open.*

eTable 7. Results by age groups, 10 to <20 years: HR and 95% CI for overall, non-syndromic and sensitivity analyses

		Analysis estimates, HR (95% CI)		
	n	Tooth agenesis	Non-syndromic tooth agenesis	Sensitivity analysis
**Any cancer**	3,646	1.11 (0.94; 1.31)	1.08 (0.91; 1.28)	-
**Lymphoid leukemias**	280	0.75 (0.37; 1.52)	0.49 (0.20; 1.19)	-
**AML**	98	1.09 (0.40; 2.97)	1.11 (0.41; 3.03)	-
**Hodgkin lymphoma**	375	1.15 (0.70; 1.90)	1.16 (0.70; 1.92)	-
**Non-Hodgkin lymphoma except Burkitt**	158	2.11 (1.17; 3.80)	2.20 (1.22; 3.96)	1.83 (0.96; 3.48)
**Astrocytomas**	207	1.30 (0.69; 2.45)	1.26 (0.65; 2.46)	-
**Osteosarcoma**	114	2.19 (1.11; 4.32)	2.32 (1.17; 4.58)	2.07 (1.01; 4.24)
**Soft tissue and other extraosseous sarcomas**	281	0.56 (0.25; 1.26)	0.53 (0.22; 1.29)	-
**Malignant extracranial and extragonadal germ cell tumors**	100	1.37 (0.56; 3.38)	1.12 (0.41; 3.05)	-
**Malignant melanoma**	238	1.62 (0.95; 2.79)	1.64 (0.96; 2.82)	1.52 (0.87; 2.67)
**Skin carcinomas**	177	1.38 (0.71; 2.71)	0.97 (0.43; 2.18)	-

Non-syndromic tooth agenesis: Re-analysis after exclusion of individuals with known genetic syndromes

Sensitivity analysis: Re-analysis after further exclusion of cases where the date of tooth agenesis diagnosis came after the date of cancer diagnosis, and tooth agenesis was registered in the hospital system only (ICD codes obtained from the Danish National Patient Registry)

Abbreviations: HR, hazard ratio; CI, confidence interval; n, number of cancer cases in age group; AML, acute myeloid leukemia

© 2024 Eiset SE et al. *JAMA Network Open.*

eTable 8. Results by age groups, 20 to <30 years: HR and 95% CI for overall, non-syndromic and sensitivity analyses

		Analysis estimates, HR (95% CI)		
	n	Tooth agenesis	Non-syndromic tooth agenesis	Sensitivity analysis
**Any cancer**	9,790	1.04 (0.93; 1.17)	1.03 (0.92; 1.16)	-
**Hodgkin lymphoma**	413	1.32 (0.80; 2.18)	1.33 (0.81; 2.20)	-
**Non-Hodgkin lymphoma except Burkitt**	182	1.16 (0.51; 2.62)	1.19 (0.53; 2.68)	-
**Pituitary adenomas and carcinomas**	242	1.01 (0.47; 2.13)	1.03 (0.49; 2.18)	-
**Soft tissue and other extraosseous sarcomas**	287	0.84 (0.40; 1.78)	0.67 (0.28; 1.64)	-
**Malignant extracranial and extragonadal germ cell tumors**	463	0.69 (0.36; 1.34)	0.70 (0.36; 1.36)	-
**Malignant gonadal germ cell tumors, males**	1,036	1.08 (0.74; 1.57)	0.98 (0.66; 1.46)	-
**Thyroid carcinomas**	252	1.13 (0.56; 2.28)	1.15 (0.57; 2.32)	-
**Malignant melanoma**	1,345	0.97 (0.70; 1.34)	0.99 (0.71; 1.37)	-
**Skin carcinomas**	871	1.38 (0.97; 1.97)	1.36 (0.95; 1.95)	-
**Carcinomas of appendix**	87	2.54 (1.03; 6.24)	2.57 (1.04; 6.32)	2.57 (1.04; 6.32)
**Carcinomas of breast, females**	<255	1.10 (0.54; 2.23)	1.12 (0.55; 2.26)	-
**Carcinomas of cervix uteri, females**	2,614	0.76 (0.59; 0.98)	0.77 (0.60; 0.99)	-
**Carcinomas of bladder**	69	3.35 (1.35; 8.30)	3.39 (1.37; 8.39)	3.39 (1.37; 8.39)

Non-syndromic tooth agenesis: Re-analysis after exclusion of individuals with known genetic syndromes

Sensitivity analysis: Re-analysis after further exclusion of cases where the date of tooth agenesis diagnosis came after the date of cancer diagnosis, and tooth agenesis was registered in the hospital system only (ICD codes obtained from the Danish National Patient Registry)

Abbreviations: HR, hazard ratio; CI, confidence interval; n, number of cancer cases in age group

© 2024 Eiset SE et al. *JAMA Network Open.*

eTable 9. Results by age groups, 30 to <40 years: HR and 95% CI for overall, non-syndromic and sensitivity analyses

		Analysis estimates, HR (95% CI)		
	n	Tooth agenesis	Non-syndromic tooth agenesis	Sensitivity analysis
**Any cancer**	8,555	1.20 (1.02; 1.40)	1.20 (1.02; 1.41)	-
**Malignant gonadal germ cell tumors, males**	628	1.58 (0.93; 2.69)	1.62 (0.95; 2.75)	-
**Thyroid carcinomas**	234	1.37 (0.56; 3.32)	1.39 (0.57; 3.36)	-
**Malignant melanoma**	1,228	1.75 (1.24; 2.47)	1.78 (1.26; 2.52)	1.78 (1.26; 2.52)
**Skin carcinomas**	1,890	1.13 (0.80; 1.61)	1.15 (0.81; 1.63)	-
**Carcinomas of colon and rectum**	192	2.81 (1.38; 5.71)	2.87 (1.41; 5.83)	2.87 (1.41; 5.83)
**Carcinomas of breast, females**	916	1.09 (0.68; 1.77)	1.12 (0.69; 1.81)	-
**Carcinomas of cervix uteri, females**	1,135	0.66 (0.39; 1.12)	0.67 (0.40; 1.14)	-

Non-syndromic tooth agenesis: Re-analysis after exclusion of individuals with known genetic syndromes

Sensitivity analysis: Re-analysis after further exclusion of cases where the date of tooth agenesis diagnosis came after the date of cancer diagnosis, and tooth agenesis was registered in the hospital system only (ICD codes obtained from the Danish National Patient Registry)

Abbreviations: HR, hazard ratio; CI, confidence interval; n, number of cancer cases in age group.

© 2024 Eiset SE et al. *JAMA Network Open.*

eFigure. Hazard Ratio (HRs) and 95% CIs for Associations Between Congenital Tooth Agenesis and Any and Specific Cancers in Patients Aged 10 to Younger Than 40 Years

Cancer type	Age group	n	HR (95% CI)
**Any cancer**	10 to <20 years	3,646	1.11 (0.94−1.31)
			1.08 (0.91−1.28)
	20 to <30 years	9,790	1.04 (0.93−1.17)
			1.03 (0.92−1.16)
	30 to <40 years	8,555	1.20 (1.02−1.40)
			1.20 (1.02−1.41)
**Lymphoid leukemias**	10 to <20 years	280	0.75 (0.37−1.52)
			0.49 (0.20−1.19)
	20 to <30 years	76	NA
			NA
	30 to <40 years	34	NA
			NA
**AML**	10 to <20 years	98	1.09 (0.40−2.97)
			1.11 (0.41−3.03)
	20 to <30 years	78	NA
			NA
	30 to <40 years	40	NA
			NA
**Hodgkin lymphoma**	10 to <20 years	375	1.15 (0.70−1.90)
			1.16 (0.70−1.92)
	20 to <30 years	413	1.32 (0.80−2.18)
			1.33 (0.81−2.20)
	30 to <40 years	114	NA
			NA
**Non−Hodgkin lymphoma***	10 to <20 years	158	2.11 (1.17−3.80)
			2.20 (1.22−3.96)
	20 to <30 years	182	1.16 (0.51−2.62)
			1.19 (0.53−2.68)
	30 to <40 years	167	NA
			NA
**Astrocytomas**	10 to <20 years	207	1.30 (0.69−2.45)
			1.26 (0.65−2.46)
	20 to <30 years	161	NA
			NA
	30 to <40 years	125	NA
			NA
**Pituitary adenomas and carcinomas**	10 to <20 years	71	NA
			NA
	20 to <30 years	242	1.01 (0.47−2.13)
			1.03 (0.49−2.18)
	30 to <40 years	166	NA
			NA
**Osteosarcoma**	10 to <20 years	114	2.19 (1.11−4.32)
			2.32 (1.17−4.58)
	20 to <30 years	36	NA
			NA
	30 to <40 years	<10	NA
			NA
**Extraosseous sarcomas**	10 to <20 years	281	0.56 (0.25−1.26)
			0.53 (0.22−1.29)
	20 to <30 years	287	0.84 (0.40−1.78)
			0.67 (0.28−1.64)
	30 to <40 years	196	NA
			NA
**Germ cell tumors****	10 to <20 years	100	1.37 (0.56−3.38)
			1.12 (0.41−3.05)
	20 to <30 years	463	0.69 (0.36−1.34)
			0.70 (0.36−1.36)
	30 to <40 years	233	NA
			NA
**Gonadal germ cell tumors (males)**	10 to <20 years	195	NA
			NA
	20 to <30 years	1,036	1.08 (0.74−1.57)
			0.98 (0.66−1.46)
	30 to <40 years	628	1.58 (0.93−2.69)
			1.62 (0.95−2.75)
**Thyroid carcinomas**	10 to <20 years	92	NA
			NA
	20 to <30 years	252	1.13 (0.56−2.28)
			1.15 (0.57−2.32)
	30 to <40 years	234	1.37 (0.56−3.32)
			1.39 (0.57−3.36)
**Malignant melanoma**	10 to <20 years	238	1.62 (0.95−2.79)
			1.64 (0.96−2.82)
	20 to <30 years	1,345	0.97 (0.70−1.34)
			0.99 (0.71−1.37)
	30 to <40 years	1,228	1.75 (1.24−2.47)
			1.78 (1.26−2.52)
**Skin carcinomas**	10 to <20 years	177	1.38 (0.71−2.71)
			0.97 (0.43−2.18)
	20 to <30 years	871	1.38 (0.97−1.97)
			1.36 (0.95−1.95)
	30 to <40 years	1,890	1.13 (0.80−1.61)
			1.15 (0.81−1.63)
**Carcinomas of colon and rectum**	10 to <20 years	13	NA
			NA
	20 to <30 years	110	NA
			NA
	30 to <40 years	192	2.81 (1.38−5.71)
			2.87 (1.41−5.83)
**Carcinomas of appendix**	10 to <20 years	97	NA
			NA
	20 to <30 years	87	2.54 (1.03−6.24)
			2.57 (1.04−6.32)
	30 to <40 years	65	NA
			NA
**Carcinomas of breast (females)**	10 to <20 years	<10	NA
			NA
	20 to <30 years	<255	1.10 (0.54−2.23)
			1.12 (0.55−2.26)
	30 to <40 years	916	1.09 (0.68−1.77)
			1.12 (0.69−1.81)
**Carcinomas of cervix uteri (females)**	10 to <20 years	38	NA
			NA
	20 to <30 years	2,614	0.76 (0.59−0.98)
			0.77 (0.60−0.99)
	30 to <40 years	1,135	0.66 (0.39−1.12)
			0.67 (0.40−1.14)
**Carcinomas of bladder*****	10 to <20 years	13	NA
			NA
	20 to <30 years	69	3.35 (1.35−8.30)
			3.39 (1.37−8.39)
	30 to <40 years	75	NA
			NA

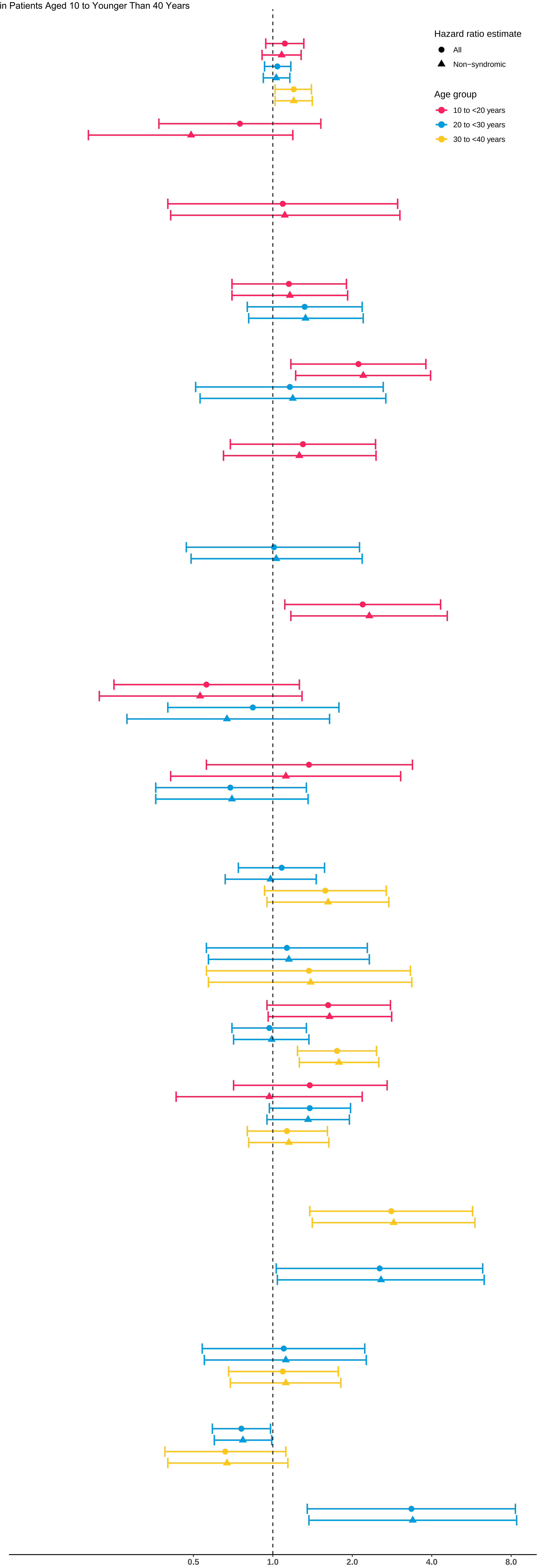

© 2024 Eiset SE et al. *JAMA Network Open.*
