# Supplementary material for: Congenital Tooth Agenesis and Risk of Early-Onset Cancer
Source: JAMA Netw Open. 2024 Mar 15;7(3):e240365. doi: 10.1001/jamanetworkopen.2024.0365 (PMC10943407; doi:10.1001/jamanetworkopen.2024.0365)
Supplement: Supplement 2. — Data Sharing Statement [file jamanetwopen-e240365-s002.pdf]

Data Sharing Statement

Eiset. Congenital Tooth Agenesis and Risk of Early-Onset Cancer. *JAMA Netw Open*. Published March 15, 2024. doi:10.1001/jamanetworkopen.2024.0365

Data
Data available: No

Additional Information
Explanation for why data not available: In accordance with Danish law and European General Data Protection Regulation, the data from this study will not be shared. Access to the data requires authorisation from the Danish Health Data Authority.
